# Role of Toll Like Receptor 4 in Alzheimer’s Disease

**DOI:** 10.3389/fimmu.2020.01588

**Published:** 2020-08-26

**Authors:** Maria Calvo-Rodriguez, Carmen García-Rodríguez, Carlos Villalobos, Lucía Núñez

**Affiliations:** ^1^Alzheimer Research Unit, Department of Neurology, Massachusetts General Hospital and Harvard Medical School, Boston, MA, United States; ^2^Instituto de Biología y Genética Molecular (IBGM), Universidad de Valladolid and Consejo Superior de Investigaciones Científicas (CSIC), Valladolid, Spain; ^3^Departamento de Bioquímica y Biología Molecular y Fisiología, Facultad de Medicina, Universidad de Valladolid, Valladolid, Spain

**Keywords:** TLR4, Alzheimer’s disease, calcium, amyloid beta oligomers, aging, hippocampal neurons

## Abstract

Long-term evidence has confirmed the involvement of an inflammatory component in neurodegenerative disorders including Alzheimer’s disease (AD). This view is supported, in part, by data suggesting that selected non-steroidal anti-inflammatory drugs (NSAIDs) provide protection. Additionally, molecular players of the innate immune system have recently been proposed to contribute to these diseases. Toll-like receptors (TLRs) are transmembrane pattern-recognition receptors of the innate immune system that recognize different pathogen-derived and tissue damage-related ligands. TLR4 mediated signaling has been reported to contribute to the pathogenesis of age-related neurodegenerative diseases, including AD. Although the pathophysiology of AD is not clear, soluble aggregates (oligomers) of the amyloid β peptide (Aβo) have been proven to be key players in the pathology of AD. Among others, Aβo promote Ca^2+^ entry and mitochondrial Ca^2+^ overload leading to cell death in neurons. TLR4 has recently been found to be involved in AD but the mechanisms are unclear. Our group recently reported that lipopolysaccharide (LPS), a TLR4 receptor agonist, increases cytosolic Ca^2+^ concentration leading to apoptosis. Strikingly, this effect was only observed in long-term cultured primary neurons considered a model of aging neurons, but not in short-term cultured neurons resembling young neurons. These effects were significantly prevented by pharmacological blockade of TLR4 receptor signaling. Moreover, TLR4 expression in rat hippocampal neurons increased significantly in aged neurons *in vitro*. Therefore, molecular patterns associated with infection and/or brain cell damage may activate TLR4 and Ca^2+^ signaling, an effect exacerbated during neuronal aging. Here, we briefly review the data regarding the involvement of TLR4 in AD.

## Toll Like Receptors and Disease

Toll-like receptors (TLRs) are innate immune receptors specialized in the detection of conserved molecular patterns present in pathogens, the so-called PAMP, and self-derived molecules released upon tissue damage, referred to as DAMP (1). The TLR family, which belongs to the type I membrane glycoproteins, is comprised of 10 members in humans, and 12 in mice (2). TLR4 was the first TLR identified in humans, which senses lipopolysaccharide (LPS)—a major component of the outer membrane of Gram-negative bacteria—which exhibits potent immuno-stimulatory activity (3). TLR4 also recognizes DAMPs released upon tissue injury, i.e., high-mobility group box 1 (HMGB1), heat-shock proteins, reactive oxygen intermediates, and extracellular matrix breakdown products (4).

TLRs recognizing bacterial and fungal components (TLR 1, 2, 4, 5, 6) are expressed on the cell surface, while sensors of viral and nucleic acids (TLR 3, 7, 8, 9, 10, 11, 12, and 13) are localized within endosomal compartments, where TLR4 can also be translocated (1, 2). The association of TLRs with their specific ligands initiates intracellular signaling routes through the adaptor MyD88, except for TLR3 that signals *via* TRIF. This culminates in the induction of pro-inflammatory molecules *via* NF-κB activation or antiviral molecules *via* interferon regulatory factor routes (3, 5). Aberrant TLR activation has been associated with chronic inflammation and disease (6). A significant amount of evidence associates TLRs to several diseases, i.e., sepsis, asthma, autoimmune diseases, cancer, diabetes, intestinal disorders, cardiovascular diseases, and neurodegenerative disorders (2, 6, 7).

In the nervous system, TLRs are expressed in several cell types including neurons and glia (8), where they sense DAMPs released by undifferentiated or necrotic/injured cells. TLR activity has been associated with several neurodegenerative diseases, including stroke, amyotrophic lateral sclerosis, Parkinson’s disease, and Alzheimer’s disease (AD) (9).

## TLR4 and Alzheimer’s Disease

TLR4 is believed to mediate the neurotoxic actions of DAMPs associated with neuronal damage involved in AD. In fact, increasing evidence associates TLR4 with neuronal plasticity (10, 11) and AD (12). For instance, Fujita and colleagues have reported that HMGB1, a prototypic DAMP released from necrotic or hyperexcitatory neurons, induces neurite degeneration *via* TLR4 (13). The study showed that myristoylated alanine-rich C-kinase substrate (MARCKS), a submembrane protein involved in the actin network stability, is phosphorylated at Ser46 well before aggregation of the amyloid β peptide (Aβ), and this effect is sustained during the course of AD both in human and mouse models of the disease. HMGB1 released from necrotic or hyperexcitatory neurons binds to TLR4 and activates MAP kinases, inducing MARCKS phosphorylation leading to neurite degeneration, one of the classic hallmarks of AD pathology. Strikingly, subcutaneous injection of an antibody against HMGB1 prevented neurite degeneration and reversed cognitive loss, even in the presence of Aβ plaques (13). This study suggests a critical involvement of TLR4 in the effects of DAMPs like HMGB1, which acts as an essential pathogenic molecule in AD.

TLR4 is also considered to be one of the key receptors involved in the microglial innate immune system, since it could be involved in the production of pro-inflammatory cytokines in AD. Consistent with this concept, gene profile analysis of post-mortem human brains revealed an increased expression of TLR4, TNF, and IL-6 genes in the frontal cortex of AD patients relative to age-matched samples (14). Moreover, in the entorhinal cortex lesioned mouse, an experimental model of hippocampal deafferentation without amyloidosis, mimicking one of the first neuronal losses observed in AD, *tlr4* and *il-1b* genes were overexpressed during the deafferentation phase but not during the process of reinnervation. Therefore, TLR4 dependent modulation of cytokines could be differentially regulated by either Aβ plaques or by deafferentation processes (14).

TLR4 activation may also contribute to AD by blocking anti-inflammatory pathways. In particular, TLR4 and TREM2 (triggering receptor expressed on myeloid cells 2, which holds an anti-inflammatory role in the brain) may be a link between AD and systemic inflammation, a process generally having deleterious effects on AD progression (15). This view is supported by data obtained from APPswe/PSEN1ΔE9 (APP/PS1) mice, a mouse model of cerebral amyloidosis, which showed increased expression of both TLR4 and TREM2 in the cortex at the gene and protein levels (15). LPS treatment further aggravated cognitive impairment in these mice, implying that superimposition of systemic inflammation to familial AD may accelerate AD progression. Interestingly, after treatment of these mice with LPS, *tlr4* gene expression remained up-regulated, while *trem2* gene was down-regulated (15). These data suggest that the inhibitory effect of TREM2 on inflammation could be downregulated by TLR4 activation, resulting in inflammation and apoptosis in the cortex of APP/PS1 mice without changes in the Aβ levels.

TLR4 has also recently been linked to memory loss mediated by Aβ oligomers (Aβo) in AD. A single intracerebroventricular injection of Aβo in C57BL/6J naïve mice substantially impaired their recognition memory. Interestingly, it also activated glial cells, resulting in the enhanced expression of pro-inflammatory cytokines. Anti-inflammatory drugs prevented the memory impairment induced by the oligomers. In addition, cyanobacterial LPS (Cyp)—a specific TLR4 receptor antagonist—eliminated the deleterious effects of Aβo on memory. Aβo had no effect either on memory or glia activation in TLR4 knockout mice, supporting the involvement of TLR4 in the noxious effects (16). Collectively, these data suggest that Aβo may not only act directly on synapses, but may also impact the immune system, with TLR4 playing a major role.

A critical role of TLR4 in AD is also supported by recent data showing that LPS, the archetypal TLR4 agonist, was detected in brain lysates from the hippocampus and neocortex of post-mortem AD brains (17). LPS levels in AD brains were found to be two to three folds larger than age-matched control cases. Strikingly, in some cases of advanced AD, there was even a 26-fold larger level of LPS over control. The authors of the study suggest that LPS from microbiota and/or bacterial infections in the body may accumulate in the brain, contributing to AD. Consistently, it has been shown that LPS is able to induce memory impairment in rats. According to these data, Zakaria et al. have recently revised and proposed rats injected with LPS as a novel animal model of AD (18).

## TLR4 and Calcium Signaling in Alzheimer’s Disease

Strong evidence supports the involvement of intracellular Ca^2+^ dyshomeostasis in aging and neurodegenerative disorders including AD (19). Our group, and others, have recently shown that long-term cultures of rat hippocampal neurons display characteristics of aged neurons (20–22). These neurons exhibit enhanced susceptibility to neuronal cell death induced by either neurotoxins such as the glutamate receptor agonist NMDA (20, 23) or oligomers of the amyloid β peptide 1-42 (24). These effects are due to age-related changes in the expression of NMDA receptors resulting in an enhanced rise in the cytosolic Ca^2+^ levels induced by the neurotoxin, and in a subsequent mitochondrial Ca^2+^ overload and apoptosis (23–25). These changes have also been related to the remodeling of Ca^2+^ homeostasis associated with aging (26) and to the toxic effects of Aβo at a subcellular level (22, 27). This model of *in vitro* neuron aging has recently been used to investigate the effects of LPS on apoptosis and Ca^2+^ signaling on hippocampal neurons.

As described above, injection of LPS in rat brains promotes cognitive decline and brain damage and the rat injected with LPS has been indeed proposed as an animal model of AD (18). Interestingly, LPS induces apoptosis in rat hippocampal neurons in primary culture depending on the time of the culture. Specifically, short-term and long-term cultures of rat hippocampal neurons resembling young and aged neurons, respectively, were activated with LPS. In morphologically identified neurons, LPS treatment promoted apoptosis only in long-term cultures, but not in young ones, as analyzed using fluorescence imaging of annexin V. These effects were inhibited by the TLR4-antagonist CAY10614, indicating the involvement of TLR4 activation (28). These effects could be mediated by TLR4 expressed in glia and/or neurons. Fluorescence imaging followed by optical density analysis showed that identified neurons express TLR4 and the level of expression increases in the long-term cultured neurons. This result was further supported by double-staining immunofluorescence proving co-expression of neuronal specific markers and TLR4 (28). These data are consistent with increased levels of TLR4 expression reported in the aged human brain (10). Additionally, recent work by Hughes and collaborators (29) suggested that in a co-culture neurons-glia, the Aβo-driven neuronal cell death is mainly due to the Aβ-sensitized TLR4 signaling of glial cells (astrocytes and microglia in the co-culture) *via* autocrine/paracrine mechanism, thus proposing another mechanism for Aβ toxicity *via* TLR4.

The effects of LPS on Ca^2+^ signaling have also been investigated in cultured rat hippocampal neurons. LPS increased cytosolic Ca^2+^ concentration ([Ca^2+^]_cyt_) in rat hippocampal neurons loaded with the Ca^2+^ sensitive dye fura2. Consistently with TLR4 expression, LPS increased [Ca^2+^]_cyt_ only in long-term cultures of rat hippocampal neurons but not in the short-term cultured ones. In contrast, NMDA increased [Ca^2+^]_cyt_ both in young and aged neurons, although the effects in aged neurons were larger (28). Again, the effects of LPS on [Ca^2+^]_cyt_ were inhibited significantly by the TLR4-antagonist CAY10614, implying TLR4-mediated effects. Glial cells also displayed Ca^2+^ responses to LPS that were mostly in the form of Ca^2+^ oscillations in a small fraction (30%) of the glial cells (28).

On the contrary, glial cells did not display Ca^2+^ responses to NMDA, as previously reported (30). The effects of LPS on [Ca^2+^]_cyt_ are not mediated by NMDARs, since low concentrations of MK801, a NMDAR antagonist, prevented NMDA-induced but not LPS-mediated rises in [Ca^2+^]_cyt_. In addition, LPS treatment did not affect the expression of the NMDAR subunits, excluding the possibility that LPS may influence Ca^2+^ signaling and apoptosis by modulating expression of NMDARs (28). These data indicate that LPS promotes Ca^2+^ signaling and apoptosis in aged neurons and TLR4 contributes to these effects by NMDA receptor independent mechanisms. However, the ion channel involved in the effects remains unknown and additional studies are required to address this question.

**FIGURE 1:**
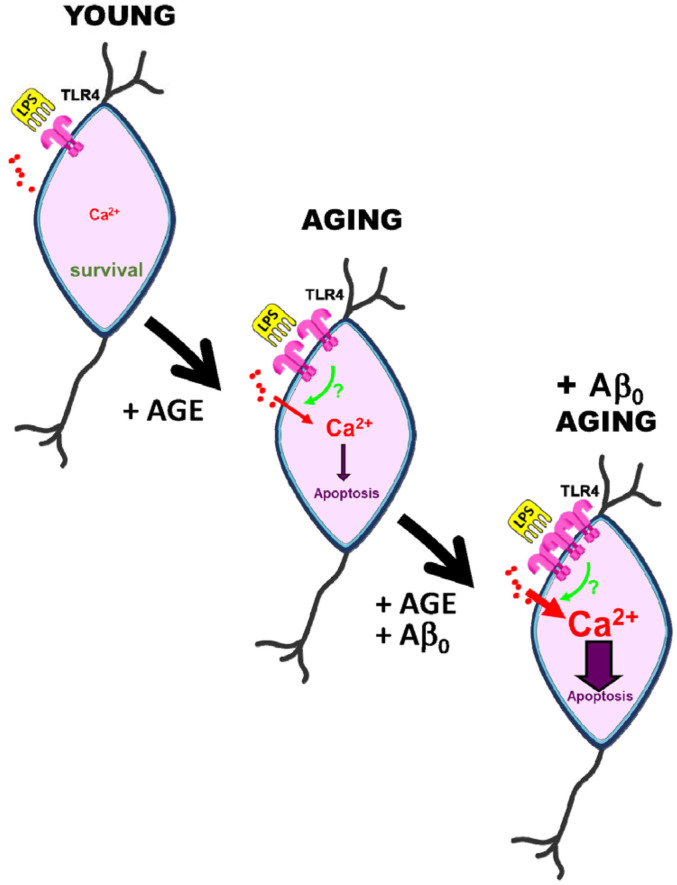
Effects of aging and amyloid oligomers on TLR4 induced Ca^2+^ signaling and death in rat hippocampal neurons aged *in vitro*. Short-term cultures of rat hippocampal neurons, resembling young neurons, display low expression of TLR4, and the TLR4 agonist LPS has no effect on cytosolic Ca^2+^ concentration or cell death. However, long-term cultured neurons resembling aged neurons display enhanced TLR4 expression, increased Ca^2+^ responses to LPS, and neuronal cell death. All three of these effects are exacerbated in aged neurons treated with amyloid β oligomers involved in Alzheimer’s disease, suggesting a crosstalk between TLR4 and amyloid β-induced Ca^2+^ signaling pathways.

The effects of amyloid oligomers on the LPS-mediated Ca^2+^ signaling and apoptosis have been addressed as well. In fact, both Ca^2+^ rises and apoptosis may be exacerbated in AD by the excess of Aβo formation. This view is supported by findings reported by Calvo-Rodriguez et al. (28) showing that co-treatment with Aβo and the TLR4 ligand LPS, potentiates Ca^2+^ responses and neuronal cell death in cultures of rat hippocampal neurons, particularly in aging. Specifically, studies performed with long-term cultured hippocampal neurons revealed that Aβo treatment potentiates the rise in [Ca^2+^]_cyt_ induced by LPS, suggesting a synergistic effect between TLR4 and Aβo involving Ca^2+^ signaling in aged neurons. Consistently, a 48 h exposure to either LPS or Aβo fails to induce apoptosis in young cultures. In contrast, the combination of LPS and Aβo significantly increased the neuronal apoptosis in young neurons, effects that were enhanced dramatically in aged neurons (28). These data indicate that Ca^2+^ signals induced by TLR4 activation and Aβo may crosstalk to enhance neuronal cell death, particularly in the aging scenario. This could be mediated by changes in the expression of TLR4 induced by Aβo. Supporting this, the treatment with Aβo induced changes in TLR4 expression in neurons depending on the time in culture. Specifically, TLR4 expression was low in young neurons and treatment with Aβo did not influence TLR4 expression, as shown by immunofluorescence. In contrast, after 2 weeks *in vitro*, when TLR4 expression is still not significantly different from young cultures, Aβo treatment increased significantly TLR4 expression. These effects were further exacerbated in neurons cultured for more than 3 weeks *in vitro*, corresponding to aged neurons. Therefore, evidence from this *in vitro* model of neuronal aging suggests that LPS promotes Ca^2+^ signaling and apoptosis in aged hippocampal neurons and that these effects are mediated by TLR4. More importantly, TLR4 expression is exacerbated by aging and the presence of Aβo (28) (Figure 1). As an *in vitro* model, the system used in this study has limitations: (i) as a model of aging, it might not reflect the complexity of *in vivo* aging in a living brain, since the effect and interaction with other cells in the brain and vessels is not considered in this model; (ii) as a model of neuroinflammation, LPS addition may be a simplistic model of recreating it, since neuroinflammation requires many other factors that are excluded here; (iii) as a model of AD, Aβ oligomers are the only element taken into account in this *in vitro* model, whereas the contribution of other factors involved in AD (tau, microglial activation, astrocytic reactivity, vessel dysfunction, etc.) remains to be explored. Nevertheless, this *in vitro* model is an excellent system to study certain precise mechanisms involving Ca^2+^ homeostasis and/or channel expression and helps in understanding the synergistic effects between Aβ and LPS on aged hippocampal neurons. Further studies *in vivo* animal models will be required to disentangle the pathology underlying this disease. Consistently, recent *in vivo* data suggest the critical role of mitochondrial Ca^2+^ overload in AD models (31).

Additionally, inhibiting TLR4 activation in AD may suppress the neuroinflammatory process in the disease. The use of chemical TLR4 antagonists as a treatment for AD might not be of high specificity. However, a broad range of therapeutic compounds inhibiting TLR4 have proven evidence of efficacy in animal models of AD (32), suggesting that therapeutic blocking of TLR4 may be a candidate therapeutic approach for AD.

## Concluding Remarks

Evidence provided in this mini review suggest the critical contribution of TLR4 in the pathogenesis of AD. TRL4 is expressed in the brain, and its expression increases with aging and the accumulation of amyloid β oligomers. DAMPs may also accumulate with aging and chronic inflammation. Therefore, the simultaneous accumulation of Aβ and DAMPs induced TLR4 activation may occur upon stress and/or brain damage, particularly in the context of aging. Increased serum levels of pro-inflammatory cytokines are often associated with aging, a chronic subclinical condition termed as inflammaging (33). Consequently, elevation in the levels of inflammatory cytokines induced by TLR4 activation may promote the accumulation of Aβ, which in turn may enhance expression of TLR4 levels, creating a damaging feedforward loop that may largely contribute to the progression of AD (34). This loop may also be further amplified by the age-associated increased expression of NMDA receptors, which could be simultaneously targeted by Aβo and perhaps by some other DAMPs. These processes may crosstalk at the level of Ca^2+^ signals induced by Aβo and TLR4 independently, particularly in the aging scenario, in which this crosstalk may contribute to brain damage during AD.

## Author Contributions

MC-R, CG-R, CV, and LN jointly wrote the mini review. All authors contributed to the article and approved the submitted version.

## Conflict of Interest

The authors declare that the research was conducted in the absence of any commercial or financial relationships that could be construed as a potential conflict of interest.
